# Metabolic, cardiovascular, neuromuscular and perceptual responses to repeated military‐specific load carriage treadmill simulations

**DOI:** 10.1002/ejsc.12154

**Published:** 2024-06-17

**Authors:** Christopher A. J. Vine, Sarah L. Coakley, Sam D. Blacker, Oliver R. Runswick, Stephen D. Myers

**Affiliations:** ^1^ Occupational Performance Research Group Institute of Applied Sciences University of Chichester Chichester UK; ^2^ Faculty of Sport, Allied Health and Performance Science St Mary's University London UK; ^3^ Institute of Psychiatry, Psychology & Neuroscience Kings College London London UK

**Keywords:** combatant, military personnel, occupational physiology, physical functional performance, physiological stress

## Abstract

Bouts of military load carriage are rarely completed in isolation; however, limited research has investigated the physiological responses to repeated load carriage tasks. Twelve civilian men (age, 28 ± 8 years; stature, 185.6 ± 5.8 cm; body mass 84.3 ± 11.1 kg and maximal oxygen uptake, 51.5 ± 6.4 mL·kg^−1^ min^−1^) attended the laboratory on two occasions to undertake a familiarisation and an experimental session. Following their familiarisation session, participants completed three bouts of a fast load carriage protocol (FLCP; ∼65 min), carrying 25 kg, interspersed with a 65‐min recovery period. Physiological strain (oxygen uptake [V̇O_2_] and heart rate [HR]) was assessed during the FLCP bouts, and physical performance assessments (weighted counter‐movement jump [wCMJ], maximal isometric voluntary contraction of the quadriceps [MIVC] and seated medicine ball throw [SMBT]) was measured pre and post each FLCP bout. A main effect for bout and measurement time was evident for V̇O_2_ and HR (both *p* < 0.001 and Ѡ^2^ = 0.103–0.816). There was no likely change in SMBT distance (*p* = 0.201 and Ѡ^2^ = 0.004), but MIVC peak force reduced by approximately 25% across measurement points (*p* < 0.001 and Ѡ^2^ = 0.133). A mean percentage change of approximately −12% from initial values was also evident for peak wCMJ height (*p* = 0.001 and Ѡ^2^ = 0.028). Collectively, these data demonstrate that repeated FLCP bouts result in an elevated physiological strain for each successive bout, along with a substantial reduction in lower body power (wCMJ and MIVC). Therefore, future research should investigate possible mitigation strategies to maintain role‐related capability.

## Highlights

- Given the progressively greater internal work rate for each successive load carriage bout (of equal external work rate), individuals responsible for load carriage planning should factor this elevated work rate into their operational planning (e.g., estimated maximal work durations).
- Elevated work rate for successive bouts should also be considered in other domains such as physical employment standards and development of working patterns.
- Group level perceptual measures appear to provide a good indication of physiological strain and therefore may provide useful information to commanders regarding the physical strain experienced by their team.

## 1 INTRODUCTION

Military load carriage is rarely completed in isolation; instead, military operators frequently complete repeated tasks in succession with little to no rest period in between. This successive completion of physical tasks could exacerbate the physiological strain placed upon personnel. However, limited studies outside of sustained operations (Lieberman et al., 2006) have investigated both the physiological strain during, and performance implications of, repeated load carriage tasks. For investigations into repeated military tasks, some physiological data have been reported; however, they have primarily focused on biomechanical (Scales et al., 2021) or cognitive performance (Giles et al., 2019). Other studies have completed prolonged load carriage tasks (∼3 h) with interspersed rest periods (10–15 min; e.g. Armstrong et al., 2022; Byrne et al., 2005; Patton et al., 1991); however, this approach may induce different physiological responses to repeated bouts, given the proximity of each marching period. As a result, there is a distinct paucity of information regarding the physiological implications of repeated military physical tasks.

Load carriage is a vital task for military operators, given that it is often critical to mission success (Knapik et al., 1996). To date, research has principally focused on factors influencing the successful completion of load carriage tasks (Drain et al., 2016; Knapik et al., 2012; Orr, 2010; van Dijk, 2007). In particular, the external load mass carried has been of key interest due to the increasing load mass that military operators are required to carry (Orr, 2010). Conversely, limited investigations have focused on load carriage tasks requiring movement speeds outside of a ‘typical’ marching speed of 4.8 km·hr^−1^. As can be observed in the new physical employment standards for the British Army (British Army, 2020), scenarios exist where mission objectives dictate that faster movement speeds are required. Previously, we described the development of a military‐specific fast load carriage protocol (FLCP) and its physiological demands (Vine et al., 2022). This protocol was designed to enhance external validity through the employment of multiple movement speeds, carrying external load mass in a representative manner and appending a simulation of a fire and maneuover task to the end of the load carriage task. Therefore, this methodology provides the ideal mechanism to further enhance external validity by investigating the repercussions of repeated load carriage bouts.

Currently, only two investigations have detailed the implications of repeated load carriage tasks (Giles et al., 2019; Scales et al., 2021). Critically, neither study had the primary focus of investigating the physiological implications of repeated load carriage tasks but instead focused on cognitive performance and biomechanical responses, respectively. In the study by Giles et al. (2019), cardiovascular strain (percent heart rate [HR] reserve) progressively increased with each load mass condition (8.8, 47.2 and 50.7 kg) with the 31 U.S. army soldiers working at a higher percentage of HR reserve during the second march compared with the first. Whilst for the Scales et al. (2021) study, 26 non‐military participants completed 2 h of load carriage, carrying either no load or 32 kg at 6.5 km·hr^−1^, on two successive days. When compared to pre‐march values on day one, the day two pre‐march V̇O_2_ was elevated by approximately 4%. Similarly, changes in V̇O_2_ across the trial were greater on day 2 compared with day 1 (∼15% vs. 9%). Given these investigations provided limited or no physiological data during the load carriage tasks and only completed two bouts; characterising the physiological responses to repeated load carriage tasks warrants further investigation.

From a military objective perspective, not only is the ability to complete the load carriage task in a strategically beneficial time frame important but military operators must also arrive with the ability to perform subsequent military tasks (Knapik et al., 1993). For example, completing a speed march to a mission objective before being able to assault an enemy position. As such, it is not only important to understand the physiological demands for a given military task but also the performance repercussions for its completion on subsequent role‐related tasks. With physical performance assessments used to quantify key physical competencies of individuals within physically demanding roles (Hauschild et al., 2017), an observed decrement in performance could suggest an attenuation in an individual's ability to successfully undertake their job role. This is broadly supported by the relationships between a combination of field‐expedient tests and common soldiering tasks detailed by Spiering et al. (2021). Previously, several authors have utilised physical performance assessments (e.g., counter‐movement jump) to assess levels of fatigue following load carriage tasks (Fallowfield et al., 2012; Knapik et al., 1997; Vine et al., 2022). We previously demonstrated a decrement in lower body performance for up to 2 h' post‐load carriage task (Vine et al., 2022). This was in line with the study in Royal Marine recruits by Fallowfield et al. (2012), whereby counter‐movement jump performance decreased following a 19.3 km march carrying 31 kg (4.3 km·h^−1^). Collectively, these data demonstrate the utility of physical performance assessments for quantifying the effects of load carriage tasks on subsequent military task performance.

Given that load carriage research to date has largely focused on isolated one‐off bouts, quantifying the implications of repeated load carriage tasks on soldiers is important to further understand the demands of military operations. Whilst these implications are likely predictable, reporting magnitudes of change would be highly valuable information for application by military end‐users (e.g., sustainability rates). The aim of this study was to investigate (1) the physiological responses to and (2) the physical repercussions of repeated bouts of military‐specific fast load carriage.

## 2 MATERIALS AND METHODS

Herein, the data are from a larger study investigating physiological and cognitive responses to repeated military load carriage. The cognitive data are reported by Vine et al. (2023). The experimental protocol comprised of a familiarisation session and an experimental session. During the familiarisation session, participants completed an unloaded treadmill walking assessment, maximal oxygen uptake (V̇O_2max_) assessment and a familiarisation to the physical performance assessments (4 kg seated medicine ball throw [SMBT], weighted counter‐movement jump [wCMJ] and maximal isometric voluntary contraction of the quadriceps [MIVC]). Participants were also familiarised with an abridged version of the FLCP. For the experimental session, participants completed the FLCP on three separate occasions with a 1:1 work–rest ratio. Pre and post each FLCP participants completed the physical performance assessments. For both sessions, participants wore a sports t‐shirt, shorts and training shoes.

Twelve physically active males, with no prior military experience, volunteered to participate (age, 28±8 years; stature, 185.6 ± 5.8 cm; body mass 84.3 ± 11.1 kg; V̇O_2max_, 51.5 ± 6.4 mL·kg^−1^ min^−1^ and body fat percentage, 14.0 ± 4.5%). Ethical approval was granted by the Institutional Review Board with data collected in accordance with the Declaration of Helsinki. Subjects were informed of the benefits and risks of the investigation prior to providing their signed consent.

Stature, body mass and body composition (measured using bioelectrical impedance) [Tanita BC—418 MA, Tanita EU, Netherlands] were recorded. Participants completed a warm‐up of 10 min unloaded walking on a motorised treadmill (HP Cosmos Saturn, HP Cosmos, Germany) with 5 minutes at 5.1 and 6.5 km·h^−1^ (1% gradient). Post‐warm up, participants were then familiarised with the three performance assessments (SMBT, wCMJ and MIVC) as described previously (Vine et al., 2022). Three maximal attempts were conducted for each physical performance assessment with 30 seconds rest between attempts.

The SMBT required the throwing of a 4‐kg medicine ball using a chest pass technique as far as possible from a seated position. The wCMJ comprised a counter movement jump, whilst wearing military webbing and a weighted vest (20 kg), with force data collected using Pasco Pasport Force platforms (PASCO, USA), sampling at 1000 Hz. The wCMJ was completed without the weapon, for safety purposes; instead, participants crossed their hands over their chest to isolate the lower body movement. The MIVC data was collected using a custom‐built chair (University of Chichester, Chichester, UK) and an s‐beam load cell (RS 250 kg, Tedea Huntleigh, Cardiff, UK), which sampled at 1000 Hz, using a PowerLab data acquisition device (AD Instruments, Oxford, UK) and a computer running Chart 4 software (V4.1.2, AD Instruments, Oxford, UK). Participants were secured in a position where their hip and knee angles were at 90° of flexion, whilst their right leg was attached to the base of the chair via the load cell and ankle cuff (Blacker et al., 2010).

Following the physical performance assessments, participants underwent a V̇O_2max_ test and subsequent verification using previously described methods (Midgley et al., 2009; Vine et al., 2022). Participants then rested for 10 minutes before completing the verification assessment again using previously described methods (Midgley et al., 2009; Vine et al., 2022). Throughout both parts of the V̇O_2max_ assessment, HR was collected continuously (V800, Polar Electro, Finland), and ∼60 s samples of expired air were collected via a mouthpiece into Douglas bags (Cranlea Human Performance Limited, Birmingham UK).

Following a recovery period, participants completed an abridged version of the FLCP. This version comprised of two 10‐min bouts of walking at 5.1 and 6.5 km·h^−1^ (1% gradient), followed by three, nine‐second shuttles at 11 km·h^−1^ (shuttles were separated by 11 s). During this familiarisation to the FLCP participants wore a belt webbing system, body armour and carried a replica assault rifle with sling (Ʃ 25.0 kg). The replica assault rifle was carried in the ‘ready position’ with the weapon slung across their chest and supported by both hands.

On the morning of the experimental trial, participants consumed a provided breakfast (carbohydrate: 34 g; fat: 5.8 g and protein: 9.6, 0.95 MJ) 1 hour before attending the laboratory having fasted for the previous 12 h. Participants then completed a standardised five‐minute warm‐up, at ∼100 W, on a cycle ergometer before completing the three performance assessments to best effort. Participants then commenced the previously described (including development) FLCP (Vine et al., 2022). The FLCP mimics movement speeds that are typical for the British Military during fast marches. It comprises of carrying the representative load of 25 kg, for 20 min at 5.1 km·h^−1^, 40 min, at 6.5 km·h^−1^, 1 min at 2.5 km·h^−1^ (1% gradient) and then undertaking 8 × 9 s shuttles, at 11 km·h^−1^ with 11 s recovery (total time 63 min 40 s).

During the FLCP, HR was recorded continuously with expired gas collected in the last 90 s of each alternate five‐minute ‘block’ (Table S1). At the end of each five‐minute ‘block’, participants were required to provide their ratings of perceived exertion (RPE; Borg, 1970), discomfort from the load (Comfort Affective Labeled Magnitude; CALM; Cardello et al., 2003) and both their thermal sensation and comfort (ASHRAE Standard, 1992; Bedford, 1936). A 150‐mL water bolus was provided to participants at four‐time points during the FLCP (Sawka et al., 2007).

On completion of the FLCP, participants were reweighed and repeated the three performance assessments to best effort. Participants rested for 10 min before being provided with a standardised snack comprising of a cereal bar and a chocolate milk drink (carbohydrate: 54.9 g; fat: 17.3 g and protein: 14.6 g, 1.86 MJ). Participants rested until they were required to re warm‐up, using the previously described warm‐up, and then completed the three performance assessments to best effort. Participants were then reweighed, and at 65 min post‐FLCP completion (1:1 work–rest ratio), participants commenced the second repeat of the FLCP. Participants completed three iterations of the above‐detailed methodology with all protocols remaining consistent. Total work duration of the trial (∼3 h) was selected to allow for direct comparisons with continuous prolonged load carriage tasks in the literature. The rest period of 65 min was selected as in the field this time would allow sufficient time for ammunition and replenishment to take place, troops to take on food and water and to be briefed for their subsequent tasking.

Statistical analysis was conducted using JASP (v0.11.1, University Amsterdam, Netherlands) with data presented as mean ± standard deviation. Using base‐2 log transformations of *p*‐values, S‐values (S) were calculated to aid clarity and interpretation of statistical estimation. Data normality were assessed using skewness and kurtosis ratios. Sphericity was also assessed, and a Greenhouse–Geisser correction applied if assumptions were violated. For physical performance assessments, a one‐way ANOVA for time was run, whilst for all other investigated variables, a two‐way repeated‐measures ANOVA was employed to investigate time, FLCP bout and interaction effects. Where F‐statistics, *p*‐values/S‐values and effect sizes, in combination indicate a likely incompatibility with the null model, *post‐hoc* pairwise comparisons, with a Holm–Bonferroni adjustment (denoted by subscript H), were made. These comparisons are presented as mean differences ± Bonferroni adjusted 95% compatibility intervals (CI_B_). For *post hoc* comparisons, Cohen's standardised means effect sizes were calculated and converted to Hedge's *g*_*z*_ to adjust for the overestimate of effect sizes associated with small sample sizes. A Friedman's test was employed for non‐parametric data with effect sizes presented using Kendall's W. Where a likely incompatibility with the null model was identified from the combination of χ^2^‐statistics, *p‐*values/S‐values and effect sizes, *post hoc* pairwise comparisons were made using Conover's test.

## 3 RESULTS

Environmental conditions for the three FLCP bouts were 13.0 ± 0.8°C WBGTi, 59 ± 9% relative humidity; 13.2 ± 0.8°C WBGTi, 57 ± 5% relative humidity and 13.4 ± 0.9°C WBGTi, 57 ± 4% relative humidity, respectively.

### 3.1 Physiological and perceptual responses

**FIGURE 1:**
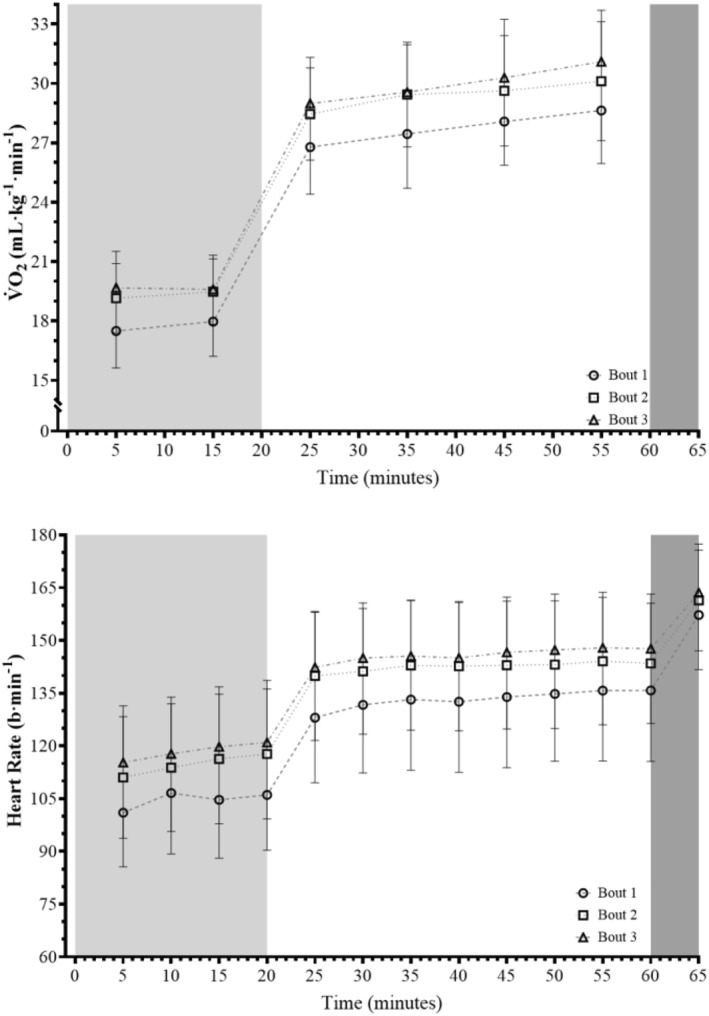
The relative V̇O_2_ and heart rate during the three Fast Load Carriage Protocol bouts. Data are presented as mean ± SD. The light grey, white and dark grey areas denote the 5.1 km·h^−1^, 6.5 km·h^−1^ and simulated fire and manoeuver portions of the protocol, respectively. Circle, square and triangle symbols denote data for bout 1, 2 and 3, respectively.

Figure 1 displays the relative V̇O_2_ for all three FLCP bouts with %V̇O_2max_ data reported in Table S2. For relative V̇O_2_ data, there was a main effect for bout and time (bout: *F*_(2, 22)_ = 73.179, *p* < 0.001, *S* > 9.97 and Ѡ^2^ = 0.141; time: *F*_(1.250, 13.751)_ = 774.886, *p* < 0.001, *S* > 9.97 and Ѡ^2^ = 0.816) but likely not an interaction effect (*F*_(3.911, 43.016)_ = 1.416, *p* = 0.183, *S* = 2.45 and Ѡ^2^ = 0.001). *Post hoc* comparisons provided evidence that relative V̇O_2_ values were greater for bouts 2 and 3 when compared with bout 1 (bouts 1 vs. 2: *t*_(2)_ = −8.896, *p*_*H*_ < 0.001, *S*_*H*_ > 9.97, *g*_*z*_ = −2.389 and 95% CI_*B*_ [−2.122 and −1.165]; bouts 1 versus 3: *t*_(2)_ = −11.548, *p*_*H*_ = 1.000, *S*_*H*_ = 0.00, *g*_*z*_ = −3.101 and 95% CI_*B*_ [−2.6122 and −1.655]) and for bout 3 when compared with bout 2 (*t*_(2)_ = −2.652, *p*_*H*_ = 0.015, *S*_*H*_ = 6.06, *g*_*z*_ = −0.712 and 95% CI_*B*_ [−0.969 and −0.011]). The average increase in relative V̇O_2_ values from bouts 1–2 and 1–3 were 9.1% and 10.9% at 5.1 km·h^−1^ and 6.1% and 8.3% at 6.5 km·h^−1^, respectively.

Figure 1 displays absolute HR for all three FLCP bouts with %HR_max_ data reported in Table S2. For HR, there was a main effect for both bout and time (bout: *F*_(2, 22)_ = 48.330, *p* < 0.001, *S* > 9.97 and Ѡ^2^ = 0.090; time: *F*_(11, 121)_ = 586.982, *p* < 0.001, *S* > 9.97 and Ѡ^2^ = 0.372), but an interaction effect was not evident (*F*_(22, 121)_ = 1.185, *p* = 0.262, *S* = 1.93 and Ѡ^2^ = 2.591e^−4^). Comparing bouts, *post hoc* analysis provided evidence that HR was greater for bouts 1 versus 2 (*t*_(2)_ = −6.966, *p*_*H*_ < 0.001, *S*_*H*_ > 9.97, *g*_*z*_ = −1.871 and 95% CI_*B*_ [−13.167 and −6.027]), 1 versus 3 (*t*_(2)_ = −9.491, *p*_*H*_ < 0.001, *S*_*H*_ > 9.97, *g*_*z*_ = −2.549 and 95% CI_*B*_ [−16.646 and −9.506]) and 2 versus 3 (*t*_(2)_ = −2.525, *p*_*H*_ = 0.019, *S*_*H*_ = 5.72, *g*_*z*_ = −0.678 and 95% CI_*B*_ [−7.049 and 0.091]). The average increase in HR at 5.1 km·h^−1^ was 9.8% for bouts 1 versus 2% and 13.6% for bouts 1 versus 3. Similarly, the average increase in HR at 5.1 km·h^−1^ was 7.4% for bouts 1 versus 2% and 10.3% for bouts 1 versus 3.

**FIGURE 2:**
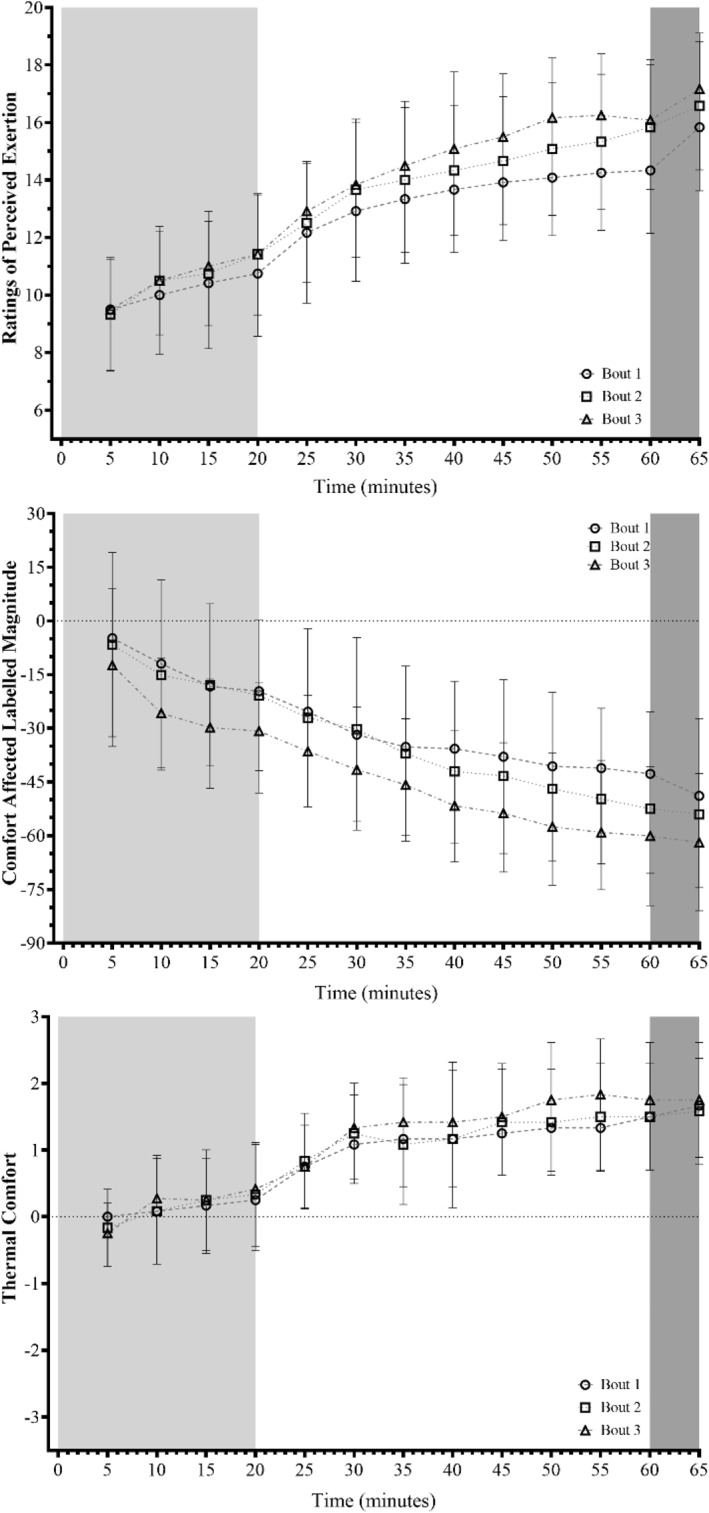
The relative Ratings of Perceived Exertion, Comfort Affective Labeled magnitude and thermal comfort scales during the three Fast Load Carriage Protocol bouts. Data are presented as mean ± SD, where light grey, white and dark grey areas denote the 5.1 km·h^−1^, 6.5 km·h^−1^ and simulated fire and manoeuver portions of the protocol, respectively. Circle, square and triangle symbols denote data for bout 1, 2 and 3, respectively.

Perceptual data are shown in Figure 2. The RPE data demonstrated a main effect of bout and time, along with a bout–time interaction effect (bout: *F*_(2, 22)_ = 7.873, *p* = 0.003, *S* = 8.38 and Ѡ^2^ = 0.047; time: *F*_(11, 121)_ = 377.726, *p* < 0.001, *S* > 9.97 and Ѡ^2^ = 0.280; interaction: *F*_(22, 121)_ = 168.492, *p* < 0.001, *S* > 9.97 and Ѡ^2^ = 0.221). Similarly, the CALM rating scores displayed a main effect for both bout and time (bout: χ^2^_(2)_ = 42.252, *p* < 0.001, *S* > 9.97 and Kendall's *W* = 3018.24; time: χ^2^_(12)_ = 263.899, *p* < 0.001, *S* > 9.97 and Kendall's *W* = −321.74). Conversely to the RPE and CALM data, the thermal comfort scale displayed no likely effect of bout (χ^2^_(2)_ = 1.841, *p* = 0.398, *S* = 1.33 and Kendall's *W* = 203.00), but a main effect of time was evident (χ^2^_(12)_ = 233.092, *p* < 0.001, *S* > 9.97 and Kendall's *W* = 27.54).

### 3.2 Performance and neuromuscular responses

**FIGURE 3:**
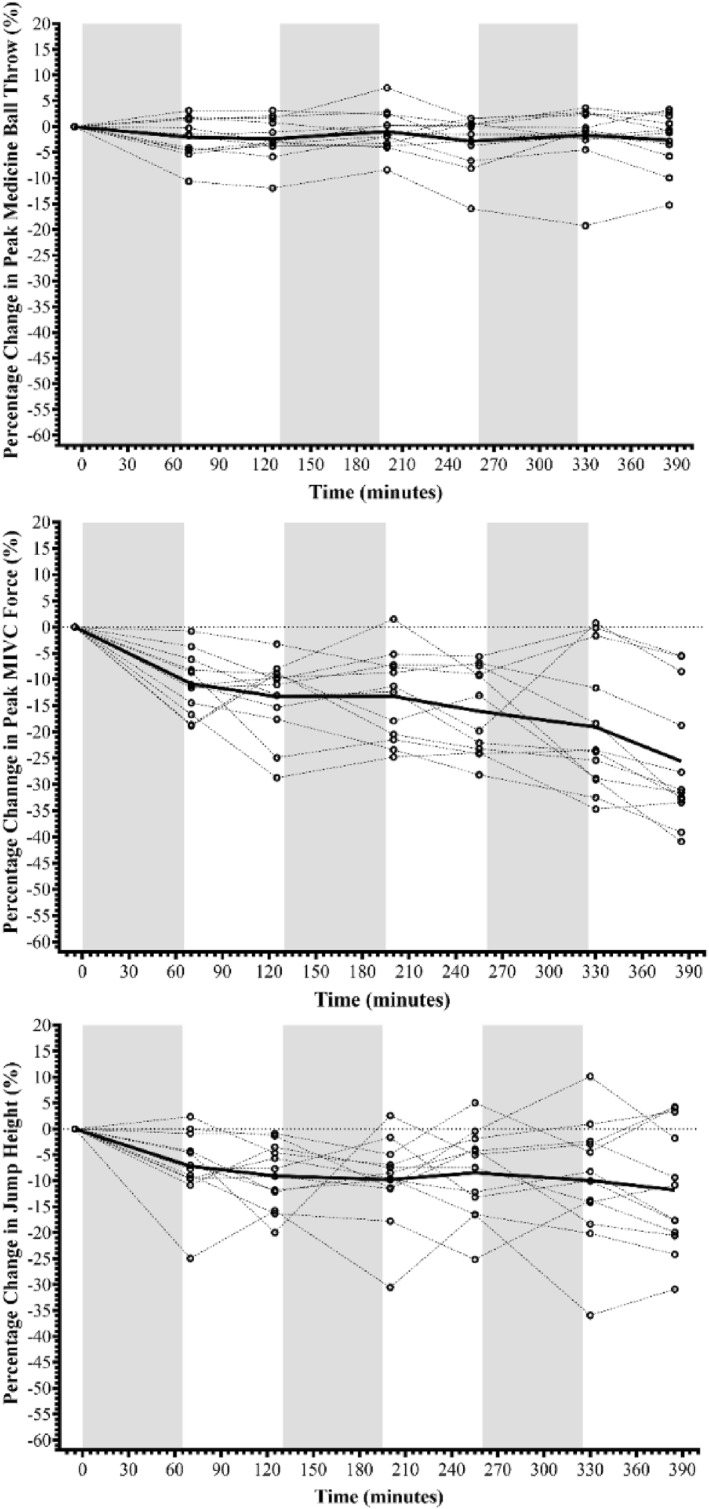
The percentage change in medicine ball throw distance, peak maximal isometric force of the quadriceps and weighted countermovement jump height across the three Fast Load Carriage Protocol bouts. Where black circles (**o**) denote individual data points with dotted lines connecting these across assessment points; thick black line (**−**) denotes the group mean average across assessment points and greyed areas denote each of the three fast load carriage protocols completed.

Percentage change data for SMBT, MIVC and wCMJ performance are shown in Figure 3 with mean and SD data for key variables presented in Table S3.

The SMBT distance likely did not differ across measurement points (*F*_(2.652, 29.174)_ = 1.660, *p* = 0.201, *S* = 2.31 and Ѡ^2^ = 0.004) with mean throw distance remaining within 0.1 m of initial values. In contrast, MIVC peak force, peak rate of force development, peak 250 ms force epoch and peak 500 ms force epoch provided evidence that values differed across time points (peak force: *F*_(2.002, 22.024)_ = 13.165, *p* < 0.001, *S* > 9.97 and Ѡ^2^ = 0.133; peak rate of force development: *F*_(6, 66)_ = 2.316, *p* = 0.043, *S* = 4.54 and Ѡ^2^ = 0.034; peak 250 ms force epoch: *F*_(1.938, 21.323)_ = 12.531, *p* < 0.001, *S* > 9.97 and Ѡ^2^ = 0.137 and peak 500 ms force epoch: *F*_(6, 66)_ = 16.851, *p* < 0.001, *S* > 9.97 and Ѡ^2^ = 0.183). At the group level, peak force reduced by approximately 200 N. *Post hoc* analysis supported a reduction in peak force with differences likely evident at all subsequent measurement points (*t*_(6)_ = 3.706–8.396, *p*_*H*_ = 0.006–<0.001, *S*_*H*_ = 7.38–>9.97 and *g*_*z*_ = 0.995–2.255). Similarly, the wCMJ variables of peak jump height and peak reactive strength index modified on force (RSI_mod_) demonstrated a likely main effect of time (peak jump height: *F*_(6, 66)_ = 4.181, *p* = 0.001, *S* = 9.97 and Ѡ ^2^ = 0.028; RSI_mod_: *F*_(6, 66)_ = 2.877, *p* = 0.015, *S* = 6.06 and Ѡ^2^ = 0.016). Whilst *post hoc* analysis did not provide evidence of a reduction in peak jump height immediately post bout 1, analysis suggested that a reduction was evident across all subsequent measurement points (*t*_(6)_ = 3.335–4.410, *p*_*H*_ = 0.024–<0.001, *S*_*H*_ = 5.38–>9.97 and *g*_*z*_ = 0.896–1.184).

## 4 DISCUSSION

Our study assessed the implications of repeated military‐specific physical activity on physiological strain and physical performance. Physiological strain increased for each successive bout of load carriage, which was largely reflected in perceptual ratings. The repeated exposure to load carriage also resulted in a progressive reduction in lower body, but not upper body, explosive power.

Both V̇O_2_ and HR exhibited substantially greater increases from bouts one to two; compared with bouts two to three, demonstrating a non‐linear increase in physiological strain and an increasing inefficiency for each successive bout. This supports Giles et al. (2019) who observed higher HRs during the second one‐hour march compared to the first during a four‐hour military scenario. In their study, group mean HR increased by ∼8%, which is a similar magnitude to the increases in V̇O_2_ and HR observed in the current study. The increase in physiological strain is likely to have important implications for military decisions regarding sustainability rates. For example, using the magnitude of V̇O_2_ drift observed by Patton et al. (1991) (13.5%), Drain et al. (2016) reported a decrease of 25% in the estimated maximum acceptable work duration for a reference load carriage task. Prior physical tasks may substantially reduce the maximum acceptable work duration even when a rest period of 1 hour is implemented. Moreover, whilst Drain et al. (2016) suggests utilising mean V̇O_2_ for a task where a V̇O_2_ drift is evident, to calculate the estimate maximum acceptable work duration, given our data demonstrating a non‐linear magnitude of increase, caution should be employed when estimating the maximum acceptable work duration for a given load carriage task, when preceded by other physical tasks. Interestingly, similar observations of progressive increases in workrate have been made by several authors during continuous three‐hour prolonged marches with interspersed 10–15 min breaks (Armstrong et al., 2022; Byrne et al., 2005; Patton et al., 1991). Thereby demonstrating similarities in the physiological implications of repeated and continuous load carriage with rest intervals. Critically, this raises the important question of where the demarcation between ‘breaks’ and ‘rest periods’ should exist. Given this similarity in physiological responses at a 1:1 work–rest ratio, future investigations should explore whether protracting the rest period between load carriage bouts would result in an attenuated increase in physiological strain.

Previously, we gathered substantial perceptual data, providing a holistic overview of the demands of the FLCP (Vine et al., 2022). In this study, this has been further enhanced through the collection of these data during all three repeated FLCP bouts. Ratings of perceived exertion were greater for bout two compared with bout one, but likely not between bouts two and three. This largely agrees with the physiological data, where the greatest magnitude of the difference was observed between bouts one and two. Plausibly the lack of statistical evidence for a difference between bouts two and three could be attributed to the large inter‐individual differences. In support of these data, Giles et al. (2019) reported RPE being greater in their second march, compared with the first, during the two marches, under medium and heavy conditions (47.2 and 50.7 kg). Importantly, in the study by Giles et al. (2019), no difference was observed between marches when carrying a light load (8.8 kg). Critically, however, their investigation only employed RPE measurements pre‐/post‐load carriage tasks. Moreover, Byrne et al. (2005) demonstrated elevated RPE ratings during three successive marches, separated by a 15‐min break, in the heat. Interestingly, in this study, a plateauing in RPE scores was evident for the final 15 min of the third march compared with continued increases in RPE at the same time points in the first and second bout, a likely positive repercussion of the spurt effect. This effect was not evident in the current investigation, purportedly due to the four‐fold greater rest period and the lack of additional heat stress. As a result, group‐level perceptual measures may provide useful information to commanders regarding the physical strain experienced by their team.

In the current study, there was no change over time in upper body explosive power assessed using the SMBT. This is similar to the outcome previously reported (Vine et al., 2022), but in contrast to previous studies, where grenade throw distance (Knapik et al., 1991) and shoulder peak torque reductions have been observed (Blacker et al., 2010); plausibly an effect of how the load was carried (webbing and body armour vs. rucksack). Decrements in both wCMJ and MIVC parameters were observed across measurement time points. Mean wCMJ jump height decreased across all time points except for immediately post the first FLCP bout. The mean change in jump height from pre‐bout one to an hour post‐bout three was approximately 3 cm. Whilst this absolute change in jump height would perhaps be considered small, given the additional load attenuating jump height already (mean initial jump was 24 cm), these jump height reductions represent considerable relative attenuations in performance. There was also a reduction in RSI_Mod_ suggesting participants were prolonging their impulse generation period, which is considered less favourable for performance (McMahon et al., 2018). As mentioned previously (Vine et al., 2022), whilst data linking decrements in RSI_Mod_ and occupational/military tasks does not exist, researchers acknowledge that reductions in physical capabilities, particularly relating to power and agility, can have significant implications for personal safety and operational success (Joseph et al., 2018). Previously, we reported the greatest observed decrement in wCMJ performance two hours‐post completion of the FLCP (Vine et al., 2022). Therefore, it could be postulated that the deficit in wCMJ could have been even greater 2 hours post completion of the final FLCP bout. A strength of the current study and a possible reason for the contrasting results is the use of a weighted versus non‐weighted countermovement jump. In a study by Pihlainen et al. (2018), the authors reported a stronger association between wCMJ performance and military simulation tasks compared with an unloaded CMJ. This could be a result of the smaller variance in performance due to the load carried and is supported by the opposing outcomes in countermovement jump performance following load carriage in the studies by Fallowfield et al. (2012) and Knapik et al. (1991). In their respective studies, a reduction (0.37 ± 0.05 m vs. 0.34 ± 0.06 m) and no change (0.46 ± 0.07 m vs. 0.45 ± 0.07 m) were observed in jump height following their load carriage tasks. From an external validity perspective, this approach also provides insight into ‘real world’ performance, given that dismounted soldiers are typically required to wear external load.

In the current study, MIVC performance deficits were observed across all key parameters and broadly across all assessment time points. The magnitude of deficit in mean peak force from pre‐bout one to an hour post‐bout three was approximately 200 N or 25%. In addition to peak force, pRFD demonstrated similar trends of attenuation, although deficit magnitudes were typically ∼10% greater for pRFD when compared with peak force. Collectively, these parameters demonstrate that participants were producing less force and at a slower rate following each bout. These deficits could have substantial implications for military operators where peak force and high rates of force development are required (e.g., climbing a wall, sprinting when assaulting an enemy position). For example, it has also been demonstrated that lower movement speeds, and thereby greater exposure time, are associated with an increase in susceptibility to enemy fire during a break contact simulation (Billing et al., 2015). Moreover, muscle function decrements may elevate musculoskeletal injury risk whilst also decreasing military physical and skilled task performance (Blacker et al., 2010).

The current study has demonstrated potentially detrimental elevations in physiological strain during and decrements in physical performance post‐repeated FLCPs which may hinder occupational performance. Future research should investigate possible mitigation strategies to maintain role‐related capability.

## CONFLICT OF INTEREST STATEMENT

The authors declare that there are no conflicts of interest/competing interests.

## Supporting information

Supporting Information S1

## Data Availability

Data for this project are available at https://osf.io/etmd3/.
