# Supplementary material for: Metabolic, cardiovascular, neuromuscular and perceptual responses to repeated military‐specific load carriage treadmill simulations
Source: Eur J Sport Sci. 2024 Jun 17;24(8):1110–9. doi: 10.1002/ejsc.12154 (PMC11295085; doi:10.1002/ejsc.12154)
Supplement: Supplementary file 1 — Supporting Information S1 [file EJSC-24-1110-s001.docx]

**Supplementary Table 1.** Overview of Experimental Measures and their Timings during the Fast Load Carriage Protocol.

| Measurement | Time (minutes) | | | | | | | | | | | | | |
| --- | --- | --- | --- | --- | --- | --- | --- | --- | --- | --- | --- | --- | --- | --- |
|  | 0 | 5 | 10 | 15 | 20 | 25 | 30 | 35 | 40 | 45 | 50 | 55 | 60 | 65^*^ |
| Speed (km·h^-1^) | 0 | 5.1 | 5.1 | 5.1 | 5.1 | 6.5 | 6.5 | 6.5 | 6.5 | 6.5 | 6.5 | 6.5 | 6.5 | FM |
| Gradient (%) | 0 | 1 | 1 | 1 | 1 | 1 | 1 | 1 | 1 | 1 | 1 | 1 | 1 | 3 |
| Perceptual Scales | ✓ | ✓ | ✓ | ✓ | ✓ | ✓ | ✓ | ✓ | ✓ | ✓ | ✓ | ✓ | ✓ | ✓ |
| V̇O_2_ |  | ✓ |  | ✓ |  | ✓ |  | ✓ |  | ✓ |  | ✓ |  |  |
| HR |  | ✓ | ✓ | ✓ | ✓ | ✓ | ✓ | ✓ | ✓ | ✓ | ✓ | ✓ | ✓ | ✓ |
| WBGTi | ✓ | ✓ | ✓ | ✓ | ✓ | ✓ | ✓ | ✓ | ✓ | ✓ | ✓ | ✓ | ✓ | ✓ |
| Water Provision |  |  |  |  | ✓ |  | ✓ |  | ✓ |  |  |  | ✓ |  |

*Where: FM, Fire and Manoeuvre Speeds – see methodology for a detailed description of the treadmill speed in this section of the protocol; V̇O_2_, oxygen uptake; HR, heart rate; WBGTi, Wet Bulb Globe Temperature Indoors; Water Provision, participants were provided with 150 mL of water at set time points during the FLCP. *note this block is not 5 minutes in duration – see methodology for a detailed description of the duration of this section of the protocol.*

**Supplementary Table 2.** Physiological Responses to three Fast Load Carriage Protocol bouts (Mean ± SD).

|  | Bout Number | Measurement Time Point (minutes) | | | | | | | | | | | | |
| --- | --- | --- | --- | --- | --- | --- | --- | --- | --- | --- | --- | --- | --- | --- |
|  |  | 5 | 10 | 15 | 20 | 25 | 30 | 35 | 40 | 45 | 50 | 55 | 60 | FM |
| V̇O_2_  (%V̇O_2max_) | 1 | 33.9 ± 3.1 |  | 34.9 ± 3.1 |  | 52.0 ± 4.3 |  | 53.1 ± 3.4 |  | 54.5 ± 4.9 |  | 55.6 ± 5.1 |  |  |
|  | 2 | 37.2 ± 3.3 |  | 37.9 ± 4.1 |  | 55.2 ± 4.6 |  | 57.1 ± 4.6 |  | 57.5 ± 4.5 |  | 58.3 ± 4.3 |  |  |
|  | 3 | 38.1 ± 3.0 |  | 38.0 ± 3.3 |  | 56.3 ± 4.3 |  | 57.4 ± 4.1 |  | 58.7 ± 4.0 |  | 60.4 ± 5.0 |  |  |
| Heart Rate  (%HR_max_) | 1 | 53.8 ± 6.0 | 56.7 ± 6.9 | 55.7 ± 6.6 | 56.5 ± 6.3 | 68.2 ± 7.4 | 70.2 ± 7.8 | 70.9 ± 8.0 | 70.6 ± 7.9 | 71.3 ± 7.9 | 71.8 ± 7.3 | 72.3 ± 7.9 | 72.3 ± 8.0 | 83.9 ± 5.8 |
|  | 2 | 59.1 ± 6.8 | 60.6 ± 7.3 | 61.9 ± 7.2 | 62.7 ± 7.1 | 74.5 ± 6.7 | 75.3 ± 6.5 | 76.1 ± 6.5 | 76.0 ± 6.3 | 76.2 ± 6.3 | 76.3 ± 6.4 | 76.8 ± 6.1 | 76.5 ± 5.7 | 86.2 ± 4.7 |
|  | 3 | 61.4 ± 5.9 | 62.8 ± 6.0 | 63.8 ± 6.2 | 64.4 ± 6.6 | 75.9 ± 5.2 | 77.3 ± 5.1 | 77.6 ± 5.1 | 77.3 ± 4.9 | 78.2 ± 4.8 | 78.4 ± 5.3 | 78.9 ± 4.7 | 78.7 ± 4.8 | 87.3 ± 3.6 |

*Where, V̇O_2_, oxygen uptake; V̇O_2max_, maximum oxygen uptake; %HRmax, percentage of heart rate maximum. Greyed areas ([ ]) denote time points where measurements were not taken. Each measurement time point details the end time of each ‘block*’.

**Supplementary Table 3**. Performance and Neuromuscular Responses to three Fast Load Carriage Protocol bouts (Mean ± SD; mean difference, [95% CI_B_]).

|  | | Measurement time point | | | | | | |
| --- | --- | --- | --- | --- | --- | --- | --- | --- |
| Performance Measure | | Pre-Bout 1 | Post-Bout 1 | Pre-Bout 2 | Post-Bout 2 | Pre-Bout 3 | Post-Bout 3 | Post-Bout 3+1 |
| SMBT | Distance (m) | 4.33 ± 0.44 | 4.23 ± 0.43 | 4.22 ± 0.43 | 4.29 ± 0.51 | 4.20 ± 0.44 | 4.25 ± 0.45 | 4.21 ± 0.43 |
| MIVC | Force (N) | 818.2 ± 136.5 | 726 ± 103.6*  92.2, [13.6, 170.7] | 711.2 ± 140.5^#^  107.0, [28.4, 185.6] | 714.7 ± 166.2*  103.5, [24.9, 182.1] | 690.1 ± 148.5^#^  128.1, [49.5, 206.7] | 664.1 ± 161.5^#^  154.1, [75.6, 232.7] | 609.4 ± 148.1^#^  208.8, [130.2, 287.4] |
|  | pRFD (N·s^-1^) | 7737 ± 3644 | 6369 ± 3132  1368, [-1164, 3899] | 5568 ± 1716  2169, [-363, 4701] | 5832 ± 3864  1905, [-627, 4437] | 5914 ± 3146  1823, [-709, 4354] | 5606 ± 3658  2131, [-401, 4663] | 5035 ± 2596*  2702, [170, 5234] |
|  | 250 s Force Epoch (N) | 811.4 ± 138.5 | 714.7 ± 107.7*  96.6, [12.3, 181.0] | 702.0 ± 145.4*  109.3, [25, 193.6] | 702.0 ± 171.5*  109.4, [25, 193.7] | 679.5 ± 152^#^  131.9, [47.6, 216.2] | 651.5 ± 166.5^#^  159.8, [75.5, 244.2] | 591.9 ± 153.3^#^  219.5, [135.1, 303.8] |
|  | 500 s Force Epoch (N) | 578.1 ± 127.6 | 508 ± 86.4*  70.1, [5.9, 134.3] | 462 ± 100.6^#^  116.1, [51.9, 180.3] | 471.7 ± 145.2^#^  106.5, [42.2, 170.7] | 449.4 ± 91.9^#^  128.8, [64.6, 193.0] | 430.8 ± 122.6^#^  147.3, [83.1, 211.5] | 394.5 ± 95.5^#^  183.7, [119.5, 247.9] |
| wCMJ | Jump Height (m) | 0.24 ± 0.05 | 0.22 ± 0.04  0.02, [0.00, 0.04] | 0.22 ± 0.04*-  0.02, [0.00, 0.04] | 0.22 ± 0.05* 0.02, [0.00, 0.05] | 0.22 ± 0.04*  0.02, [0.00, 0.04] | 0.22 ± 0.05*  0.02, [0.00, 0.05] | 0.21 ± 0.05^#^  0.03, [0.01, 0.05] |
|  | RSI_Mod_ (ratio) | 0.24 ± 0.08 | 0.23 ± 0.08  0.01, [-0.02, 0.04] | 0.22 ± 0.07  0.03, [-0.01, 0.06] | 0.21 ± 0.06  0.03, [-0.01, 0.06] | 0.22 ± 0.07  0.02, [-0.01, 0.05] | 0.22 ± 0.07  0.02, [-0.01, 0.05] | 0.20 ± 0.06*  0.04, [0.00, 0.07] |

*Note: Mean difference, [95% CI_B_] compared with values Pre-Bout 1. Mean difference, [95% CI_B_] values only presented where post-hoc comparisons were calculated.* ^#^  Denotes *p_H_ < 0.001 and* ^*^ denotes *p_H_ < 0.05 when compared with values Pre-Bout 1. Where, SMBT, Seated Medicine Ball Throw; wCMJ, weighted Counter Movement Jump; MIVC, Maximal Isometric Voluntary Contraction of the quadriceps; RSI_Mod_, Reactive Strength Index Modified; pRFD, peak rate of force development; Post-Bout 3+1, measurement point one-hour post third bout.*
